# 2-h postchallenge plasma glucose predicts cardiovascular events in patients with myocardial infarction without known diabetes mellitus

**DOI:** 10.1186/1475-2840-11-93

**Published:** 2012-08-08

**Authors:** Loghman Henareh, Stefan Agewall

**Affiliations:** 1 Department of Cardiology Karolinska University Hospital, Karolinska Institute, Stockholm, Sweden; 2 Department of Cardiology, Oslo University Hospital Ullevål, Oslo University, Oslo, Norway

## Abstract

**Background and purpose:**

The incidence of cardiovascular events remains high in patients with myocardial infarction (MI) despite advances in current therapies. New and better methods for identifying patients at high risk of recurrent cardiovascular (CV) events are needed. This study aimed to analyze the predictive value of an oral glucose tolerance test (OGTT) in patients with acute myocardial infarction without known diabetes mellitus (DM).

**Methods:**

The prospective cohort study consisted of 123 men and women aged between 31–80 years who had suffered a previous MI 3–12 months before the examinations. The exclusion criteria were known diabetes mellitus. Patients were followed up over 6.03 ± 1.36 years for CV death, recurrent MI, stroke and unstable angina pectoris. A standard OGTT was performed at baseline.

**Results:**

2-h plasma glucose (HR, 1.27, 95% CI, 1.00 to 1.62; P < 0.05) and smoking (HR, 3.56, 95% CI, 1.02 to 12.38; P < 0.05) proved to be independent predictors of CV events in multivariate statistical analysis after adjustments for age, sex, total cholesterol, and other baseline characteristics.

**Conclusions:**

In this study population, with previous MI and without known DM, 2-h PG and smoking were significant predictors of CV death, recurrent MI, stroke and unstable angina pectoris, independent of baseline characteristics and medical treatment.

## Introduction

Previous studies have shown that almost two thirds of patients with cardiovascular disease suffer from abnormal glucose metabolism [1,2]. The majority of these cases are not detected by fasting plasma glucose (FPG) but with 2-h PG after OGTT [1,3]. There is evidence that postprandial hyperglycemia is an independent risk factor for atherosclerosis and has an even greater effect than fasting plasma glucose [4,5] on future events.

The pathophysiology behind the association between postprandial hyperglycemia and atherosclerosis is not fully understood. Our research group has previously reported a correlation between 2-h PG and inflammation parameters in patients with coronary artery disease (CAD) [4].

According to current understanding, hyperglycemia induces oxidative stress which, in combination with soluble advanced glycation end products (AGEs) and lipid peroxidation products leads to endothelial dysfunction and expression of inflammatory genes [6-8].

The purpose of this study was to analyze the predictive value of an OGTT in patients with acute myocardial infarction (AMI) without known DM after several years of follow-up.

## Methods

### Subjects

123 patients, men and women aged between 31–80 years with a previous acute MI, took part in the study. The inclusion criterion was hospital-diagnosed myocardial infarction that had occurred 3–12 months before the examinations. The patients were recruited from the department of Cardiology at Karolinska University Hospital Huddinge, Sweden. 90% of the participants were examined within three months of the MI. We chose three months to ensure the examination took place when patients were in a clinically stable condition. All patients who were admitted to the cardiac intensive care unit because of acute myocardial infarction were included in the study, consecutively, throughout the period 2002–2003. The exclusion criteria were known diabetes mellitus and chronic inflammatory disease. Blood samples had been taken for the control of inflammatory factors in another study. All subjects gave informed consent after written and oral information.

The Karolinska Institute ethics committee at Karolinska University Hospital Huddinge approved the study. All of the patients except five (who had moved to another city) were followed up at our outpatient clinic every six months and all CV events were recorded in a case report form (CRF). The five patients who had moved were telephoned for a clinical check-up every six months.

Acute MI was defined using the criteria of the European Society of Cardiology and the American College of Cardiology [9].

### Follow-up and cardiovascular events

All patients were followed up over 6.03 ± 1.36 years. The primary end point was defined as any of the following: death from any cause, nonfatal reinfarction or stroke, unstable angina pectoris, congestive heart failure requiring hospitalization, and coronary revascularization procedure (percutaneous coronary angioplasty or coronary artery bypass grafting). Time to first event was used as the endpoint. When revascularization procedures occurred during AMI or unstable angina, it was recorded as a single event (e.g. AMI treated with primary percutaneous angioplasty was recorded as AMI. A peripheral vascular event was defined as any increase in peripheral ischemic symptoms resulting in any peripheral revascularization procedure (percutaneous transluminal angioplasty or operation). The absence of any of these features was considered as event-free survival.

### Measurements

Venous blood was drawn after an overnight fast and five min of supine rest, to determine the plasma glucose and plasma levels of cholesterol and triglycerides using established methods. Plasma glucose concentrations at 0 and 120 min following ingestion of 75 g glucose were analyzed, using glucose oxidase technique on a Hitachi 917 system. DM and impaired glucose tolerance (IGT) were defined based on American Diabetes Association (ADA) definitions [10]. On the basis of 2-h PG alone, individuals were classified into categories of newly diagnosed diabetes, IGT and normal glucose tolerance (NGT) if their 2-h PG concentrations were ≥ 11.1, 7.8–11.0 and < 7.8 mmol/l, respectively.

Resting blood pressure was measured in the right arm after about 10 min of supine rest. Body mass index (BMI) was measured according to recommended principles. Smoking was assessed by a questionnaire.

### Echocardiography

All patients underwent a standard echocardiographic evaluation, using a 2.5 MHz transducer (System Five, GE Vingmed, Horten, Norway). The echocardiographic studies were performed with the subject in supine left lateral decubitus, after 30 minutes of rest. One physician recorded all the echocardiograms. Two-dimensional imaging of the longitudinal parasternal view was checked in order to avoid angulation of the ultrasonic beam, and consequent changes in the left ventricular shape. Left ventricular internal dimension, left ventricular posterior wall and interventricular septum thickness were measured, according to the recommendations of the American Society of Echocardiography [11]. The ejection fraction was calculated according to Simpson’s formula.

### Statistical analysis

Results are presented as means and standard deviations of the mean. All data analyses were done using Statistica for Windows software version 10.0. Mann–Whitney *U* test and X² test were performed. The Cox regression model was used to identify the predictive factors.

## Results

**Figure 1:**
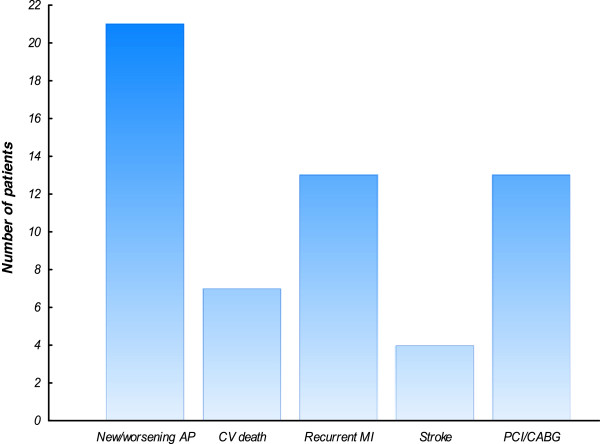
**Distribution of the various CV events in the patients with CV events.** AP= angina pectoris, CV= cardiovascular event, MI= myocardial infarction. PCI= percutaneous coronary intervention, CABG= coronary artery bypass grafting.

Table 1 shows the baseline characteristics of the study patients. There were no significant differences between the patients with CV events compared with those without CV events regarding known risk factors such as hypertension, smoking habits, cholesterol value, LVEF and current medication. During the follow-up at 6.03 ± 1.36 years, CV events had occurred in 30 patients. Figure 1 shows the distribution of the various CV events in the patients.

**Table 1:** Baseline characteristics

	**Subjects with CV event n = 30**	**Subjects without CV event n = 93**
Age (years)	62 ± 12	61 ± 10
Gender	23 (77)	71 (76)
- Male, n (%)	7 (23)	22 (24)
- Female, n (%)		
Smoking habits	6 (20)	14 (15)
- Yes, n (*%*)	4 (14)	24 (26)
- Never, n (*%*)	19 (63)	52 (56)
- Prior smoker, n (*%*)	1 (3)	3 (3)
- Moist snuff, n (*%*)		
BMI (kg/m^2^)	27 ± 4	27 ± 4
Systolic blood pressure (mmHg)	135 ± 21	138 ± 20
Diastolic blood pressure (mmHg)	77 ± 9	80 ± 9
Triglycerides, mmol/l (mean±SD)	1.7 ± 1.0	1.6 ± 1.0
Total cholesterol, mmol/l (mean±SD)	4.2 ± 0.7	4.1 ± 0.7
Heart rate, beats/min (mean±SD)	61 ± 11	58 ± 9
Fasting plasma glucose (mmol/l)	5.5 ± 1.7	5.4 ± 1.1
2-h plasma glucose (mmol/l)	8.1 ± 3.3⪵	7.5 ± 3.0
Ejection fraction (%)	49 ± 11	54 ± 7.0

*P<0.05 Mann Whitney test.

Table 2 shows previous interventions and medical treatment in the study population. Coronary angiography was performed in 92 patients. That showed one vessel disease in 37 patients, two vessel diseases in 15 patients and 3 vessel diseases in 22 patients. In 16 cases no significant coronary artery stenosis was identified.

**Table 2:** Interventions and treatment in the study group (n=123)

**Interventions**	**All subjects (n=123)**	**Subjects with CV events (n=30)**	**Subjects without CV events (n=93)**
Previous PCI, n (%)	58 (47)	13 (43)	45 (48)
Previous CABG, n (%)	20 (16)	6 (20)	14 (15)
Thrombolysis, n (%)	18 (15)	3 (10)	15 (16)
**Drugtreatment**			
Aspirin, n (%)	122 (99)	30 (100)	92 (99)
Betablocker, n (%)	111 (90)	25 (83)	86 (92)
ACE inhibitor, n (%)	31 (25)	10 (33)	21 (23)
Statin, n (%)	111 (90)	28 (93)	83 (94)

*PCI*= percutaneus coronary intervention. *CABG*= Coronary artery by-pass grafting.

*ACE*= angiotensin-converting enzyme. *CV*= Cardiovascular event.

Table 3 shows baseline characteristics of the subjects with DM, IGT and NGT. We found no statistically significant difference between the three groups with DM, IGT and NGT for the occurrence of CV events.

**Table 3:** Characteristics of the subjects with DM, IGT and NGT

	**DM (n = 13)**	**IGT (n = 29)**	**NGT (n = 68)**
Famle	3 (23%)	6 (21%)	18 (26%)
Age (year)	61 ± 11	65 ± 8*	60 ± 12
BMI (kg/m^2^)	29 ± 3^†^	27 ± 4	26 ± 3
Triglycerides (mmol/l)	2.7 ± 1.1^†,§^	1.8 ± 1.3	1.4 ± 0.7
Cholestrol (mmol/l)	4.9 ± 1.1	4.6 ± 0.8	4.6 ± 1.1
FPG (mmol/l)	7.7 ± 2.6^†,§^	5.4 ± 0.6*	4.9 ± 0.5
2-h PG (mmol/l)	14.4 ± 3.4^†,§^	9.2 ± 0.90*	5.8 ± 1.0
Systolic blood pressure (mmHg)	146 ± 25^†^	146 ± 20*	133 ± 19
Diastolic blood pressure (mmHg)	79 ± 11	82 ± 10*	78 ± 9
Cardiovascular events, n(%)	3 (23)	8 (27)	19 (28)

DM, diabetes mellitus; IGT, impaired glucose tolerance; NGT, normal glucose tolerance.

† DM vs. NGT; P < 0.05.

* IGT vs. NGT; P < 0.05.

§ DM vs. IGT; p<0.05.

Table 4 shows the Cox proportional-hazards regression model with the primary end point as outcome for some important baseline variables. In this study we have only analyzed triglycerides and total cholesterol. Both these and BMI were entered in Cox proportional-hazards regression model, but they showed no significant predictive value.

**Table 4:** Cox proportional-hazards regressions model with primary end point as outcome, including all important baseline variables

	**Age-and gender adjusted Cox regressions**			**Univariate logistic regression**		
	**B (SE)**	**HR (95% CI)**	**P value**	**B (SE)**	**OR (95% CI)**	**P value**
Age (years)	0.03 (0.03)	1.03 (0.96-1.10)	0.38	0.01 (0.03)	1.01 (0.96-1.07)	0.66
Systolic BP mmHg	0.02 (0.65)	1.02 (0.29-3.63)	0.98	-0.25 (0.65)	0.78 (0.22-2.78)	0.70
Fasting PG mmol/l	−0.81 (0.47)	0.44 (0.18–1.11)	0.08	-0.27 (0.37)	1.31 (0.37-1.59)	0.47
2h PG mmol/l	0.25 (0.12)	1.27 (1.00-1.62)	0.04	0.08 (0.09)	1.08 (0.91-1.29)	0.38
Smoking	1.27 (0.64)	3.56 (1.02-12.38)	0.04	1.34 (0.62)	3.83 (1.14-12.85)	0.03
Sex	0.12 (0.33)	1.27 (0.35-4.66)	0.72	0.03 (0.70)	1.03 (0.26-4.09)	0.96

BP= blood pressure, PG= plasma glucose, HR= hazard ratio, OR= odds ratio.

In multivariate statistical analysis and after adjustment for age, sex, total cholesterol, and other baseline characteristics, 2-h plasma glucose (HR, 1.27, 95% CI, 1.00 to 1.62; P < 0.05) and smoking (HR, 3.56, 95% CI, 1.02 to 12.38; P < 0.05) remained as independent predictors of CV events.

## Discussion

During the six-year follow-up, the incidence of cardiovascular events after myocardial infarction was 24%. Smoking and 2-h PG were two independent predictors of CV in patients with myocardial infarction. Our research group has previously reported that the prevalence of IGT and unknown DM is high in patients with ischemic heart disease [1]. This study confirms the results from previous studies [12,13] and highlights the clinical relevance of hyperglycemia 2-h post glucose challenge as independent risk factors for CV events in patients with ischemic heart disease. Several epidemiological studies have indicated that patients with pre-diabetic conditions, below the threshold for diabetes, are at higher risk for cardiovascular disease [14,15]. Thus, previous studies have shown that IGT and newly detected diabetes were risk factors for increased CV events after AMI [16,17]. However, it's still unclear whether patients with 2-h postchallenge glucose below the threshold for DM after MI are at a higher risk of CV events. This study also suggests, in line with previous reports [18,19] that 2-h PG is a better risk predictor of CV events than FPG.

Concurring with our study, Schinner et al. [20] found a high prevalence of impaired glucose metabolism in patients with coronary heart disease (CHD) assessed by coronary angiography. They found a continuous increased risk of CHD with blood glucose levels even in the subdiabetic range. However, as in our study, they found that post-prandial hyperglycaemia contributed more to CHD than fasting hyperglycemia.

The pathophysiological mechanism behind the relationship between 2-h plasma glucose and CV events is not fully understood. Previous studies have shown that there is a correlation between 2-h plasma glucose with higher levels of plasminogen activator inhibitor (PAI) [21] and high sensitive C-reactive protein as a marker for low grade inflammation [4]. In line with our study, Chu et al. [22] showed that postchallenge hyperglycemia, increased levels of pro-inflammatory markers such as tumor necrosis factor alpha (TNF-α) and nitrotyrosone time-dependently, and that these levels were associated with coronary artery disease (CAD) in patients without previous recognized diabetes.

Disturbed glucose metabolism is associated with left ventricular dysfunction and increased intima media thickness of the carotid artery [23]. Patients with IGT often develop metabolic syndrome with increased obesity. Thus the pathophysiological relationship between 2-h plasma glucose and CV events may be explained by different mechanisms.

Other investigators have suggested age, left ventricular ejection fraction, use of beta-blockers, aspirin and statins as potential predictors for long-term CV inpatients with AMI [24-27] But even after adjustment for pharmacological therapy, age and other proposed predictors, we found that 2-h post load PG and smoking were independent predictors of CV events following AMI. The observation that smoking predicts CV disease is in line with previous studies [28,29].

Smoking is an independent risk factor for all-cause mortality and cardiovascular death and is also associated with impaired glucose tolerance and increased risk of type 2 diabetes [30,31]. The pathophysiological mechanism by which smoking effects glucose intolerance and worsens clinical outcomes in diabetic patients is not fully understood. According to previous studies, smoking leads to increased insulin resistance, beta cell dysfunction, endothelial dysfunction and low-grade chronic inflammation [32].

Systolic blood pressure was also associated with the CV events; however, the relationship was significant only in univariate analysis. Previous studies have shown a correlation between high blood pressure and metabolic changes, such as impaired glucose tolerance and postchallenge hyperglycemia. The exact mechanism of this correlation remains somewhat unclear. It has been demonstrated that postprandial hyperglycemia is associated with increased oxidative stress [33,34] and endothelial dysfunction [35,36] and this would promote the development of atherosclerosis [36] and hypertension [36,37]. Furthermore, hyperglycemia is related to decreased blood flow to skeletal muscle, resulting in decreased glucose utilization [38].

### Study limitations

This study consisted of a small number of patients in a single center. Thus, despite a seemingly convincing message, our results may not reflect the real world population.

In conclusion, we show that in this study population with previous MI without known DM, 2-h plasma glucose and smoking were significant predictors of CV death, recurrent MI, stroke and unstable angina pectoris, independent of baseline characteristics and medical treatment.

### Clinical implication

Our results suggest that 2-h PG and smoking could be linked to an increased risk of CV events in patients with previous MI. An OGTTcould be added to the standard risk evaluation procedures in a hospital settings, as a potential method for preventing CV events it could be the focus of future clinical investigations.

## Abbreviations

OGTT: Oral glucose Tolerance Test; CV: Cardiovascular; MI: Myocardial Infarction; DM: Diabetes Mellitus; FPG: Fasting Plasma Glucose; CAD: Coronary Artery Disease; AGEs: Advanced glycation End products; AMI: Acute Myocardial Infarction; CRF: Case Report Form; IGT: Impaired Glucose Tolerance; NGT: Normal glucose tolerance; BMI: Body Mass Index; ADA: American Diabetes Association; CHD: Coronary Heart Disease; TNF-α: Tumor Necrosis Factor alpha; PAI: Plasminogen Activator Inhibitor.

## Competing interests

Both authors declare that they have no competing interest.

## Authors’ contribution

LH: participating in study design, data analysis, patient enrollment and writing manuscript. SA: Participating in study design, interpreted the results and revised the manuscript. Both authors read and approved the final manuscript.

## References

[B1] Henareh L Berglund M Agewall S Should oral glucose tolerance test be a routine examination after a myocardial infarction? Int J Cardiol 2004 97 1 21 24 10.1016/j.ijcard.2003.06.023 15336801

[B2] Conaway DG O'Keefe JH Reid KJ Spertus J Frequency of undiagnosed diabetes mellitus in patients with acute coronary syndrome Am J Cardiol 2005 96 3 363 365 10.1016/j.amjcard.2005.03.076 16054458

[B3] Leiter LA Ceriello A Davidson JA Hanefeld M Monnier L Owens DR Tajima N Tuomilehto J Postprandial glucose regulation: new data and new implications Clin Ther 2005 27 Suppl B S42 S56 16519037 10.1016/j.clinthera.2005.11.020

[B4] Henareh L Jogestrand T Agewall S Glucose intolerance is associated with C-reactive protein and intima-media anatomy of the common carotid artery in patients with coronary heart disease Diabet Med 2005 22 9 1212 1217 10.1111/j.1464-5491.2005.01577.x 16108851

[B5] Hanefeld M Fischer S Julius U Schulze J Schwanebeck U Schmechel H Ziegelasch HJ Lindner J Risk factors for myocardial infarction and death in newly detected NIDDM: the diabetes intervention study, 11-year follow-up Diabetologia 1996 39 12 1577 1583 10.1007/s001250050617 8960845

[B6] Giugliano D Ceriello A Paolisso G Oxidative stress and diabetic vascular complications Diabetes Care 1996 19 3 257 267 10.2337/diacare.19.3.257 8742574

[B7] Schmidt AM Yan SD Wautier JL Stern D Activation of receptor for advanced glycation end products: a mechanism for chronic vascular dysfunction in diabetic vasculopathy and atherosclerosis Circ Res 1999 84 5 489 497 10.1161/01.RES.84.5.489 10082470

[B8] Crisby M Kublickiene K Henareh L Agewall S Circulating levels of autoantibodies to oxidized low-density lipoprotein and C-reactive protein levels correlate with endothelial function in resistance arteries in men with coronary heart disease Heart Vessels 2009 24 2 90 95 10.1007/s00380-008-1089-y 19337791

[B9] Thygesen K Alpert JS White HD Jaffe AS Apple FS Galvani M Katus HA Newby LK Ravkilde J Chaitman B Universal definition of myocardial infarction Circulation 2007 116 22 2634 2653 10.1161/CIRCULATIONAHA.107.187397 17951284

[B10] Diagnosis and classification of diabetes mellitus Diabetes Care 2010 33 Suppl 1 S62 S69 20042775 10.2337/dc10-S062 PMC2797383

[B11] Schiller NB Shah PM Crawford M DeMaria A Devereux R Feigenbaum H Gutgesell H Reichek N Sahn D Schnittger I Recommendations for quantitation of the left ventricle by two-dimensional echocardiography. american society of echocardiography committee on standards, subcommittee on quantitation of two-dimensional echocardiograms J Am Soc Echocardiogr 1989 2 5 358 367 2698218 10.1016/s0894-7317(89)80014-8

[B12] Barr EL Zimmet PZ Welborn TA Jolley D Magliano DJ Dunstan DW Cameron AJ Dwyer T Taylor HR Tonkin AM Risk of cardiovascular and all-cause mortality in individuals with diabetes mellitus, impaired fasting glucose, and impaired glucose tolerance: the australian diabetes, obesity, and lifestyle study (AusDiab) Circulation 2007 116 2 151 157 10.1161/CIRCULATIONAHA.106.685628 17576864

[B13] Meigs JB Nathan DM D'Agostino RB Wilson PW Fasting and postchallenge glycemia and cardiovascular disease risk: the Framingham offspring study Diabetes Care 2002 25 10 1845 1850 10.2337/diacare.25.10.1845 12351489

[B14] Tominaga M Eguchi H Manaka H Igarashi K Kato T Sekikawa A Impaired glucose tolerance is a risk factor for cardiovascular disease, but not impaired fasting glucose. The funagata diabetes study Diabetes care 1999 22 6 920 924 10.2337/diacare.22.6.920 10372242

[B15] Glucose tolerance and mortality: comparison of WHO and American Diabetes Association diagnostic criteria The DECODE study group. European Diabetes Epidemiology Group. Diabetes Epidemiology: Collaborative analysis Of Diagnostic criteria in Europe Lancet 1999 354 9179 617 621 10466661

[B16] Bartnik M Malmberg K Norhammar A Tenerz A Ohrvik J Ryden L Newly detected abnormal glucose tolerance: an important predictor of long-term outcome after myocardial infarction Eur Heart J 2004 25 22 1990 1997 10.1016/j.ehj.2004.09.021 15541834

[B17] Tamita K Katayama M Takagi T Akasaka T Yamamuro A Kaji S Morioka S Kihara Y Impact of newly diagnosed abnormal glucose tolerance on long-term prognosis in patients with acute myocardial infarction Circulation journal: official journal of the Japanese circulation society 2007 71 6 834 841 10.1253/circj.71.834 17526977

[B18] Qiao Q Pyorala K Pyorala M Nissinen A Lindstrom J Tilvis R Tuomilehto J Two-hour glucose is a better risk predictor for incident coronary heart disease and cardiovascular mortality than fasting glucose Eur Heart J 2002 23 16 1267 1275 10.1053/euhj.2001.3113 12175663

[B19] Kitada S Otsuka Y Kokubu N Kasahara Y Kataoka Y Noguchi T Goto Y Kimura G Nonogi H Post-load hyperglycemia as an important predictor of long-term adverse cardiac events after acute myocardial infarction: a scientific study Cardiovasc Diabetol 2010 9 75 10.1186/1475-2840-9-75 21070650 PMC2996353

[B20] Schinner S Futh R Kempf K Martin S Willenberg HS Schott M Dinh W Scherbaum WA Lankisch M A progressive increase in cardiovascular risk assessed by coronary angiography in non-diabetic patients at sub-diabetic glucose levels Cardiovasc Diabetol 2011 10 56 10.1186/1475-2840-10-56 21702911 PMC3142488

[B21] Henkel E Kohler C Temelkova-Kurktschiev T Hanefeld M Predictors of abnormal glucose tolerance in persons at risk of type 2 diabetes: the RIAD study Dtsch Med Wochenschr 2002 127 18 953 957 10.1055/s-2002-26731 11987015

[B22] Chu CS Lee KT Cheng KH Lee MY Kuo HF Lin TH Su HM Voon WC Sheu SH Lai WT Postchallenge responses of nitrotyrosine and TNF-alpha during 75-g oral glucose tolerance test are associated with the presence of coronary artery diseases in patients with prediabetes Cardiovasc Diabetol 2012 11 21 10.1186/1475-2840-11-21 22397368 PMC3316140

[B23] Henareh L Lind B Brodin LA Agewall S Disturbed glucose metabolism is associated with left ventricular dysfunction using tissue doppler imaging in patients with myocardial infarction Clin Physiol Funct Imaging 2007 27 1 60 66 10.1111/j.1475-097X.2007.00717.x 17204040

[B24] Collaborative meta-analysis of randomised trials of antiplatelet therapy for prevention of death, myocardial infarction, and stroke in high risk patients BMJ 2002 324 7329 71 86 10.1136/bmj.324.7329.71 11786451 PMC64503

[B25] Metoprolol in acute myocardial infarction (MIAMI) A randomised placebo-controlled international trial. The MIAMI trial research group Eur Heart J 1985 6 3 199 226 2863148

[B26] Yusuf S Sleight P Pogue J Bosch J Davies R Dagenais G Effects of an angiotensin-converting-enzyme inhibitor, ramipril, on cardiovascular events in high-risk patients. The Heart Outcomes Prevention Evaluation Study Investigators N Engl J Med 2000 342 3 145 153 10639539 10.1056/NEJM200001203420301

[B27] Randomised trial of cholesterol lowering in 4444 patients with coronary heart disease: the Scandinavian Simvastatin survival study (4 S) Lancet 1994 344 8934 1383 1389 7968073

[B28] Al-Delaimy WK Willett WC Manson JE Speizer FE Hu FB Smoking and mortality among women with type 2 diabetes: The nurses' health study cohort Diabetes Care 2001 24 12 2043 2048 10.2337/diacare.24.12.2043 11723080

[B29] Fuller JH Stevens LK Wang SL Risk factors for cardiovascular mortality and morbidity: the WHO mutinational study of vascular disease in diabetes Diabetologia 2001 44 Suppl 2 S54 S64 11587051 10.1007/pl00002940

[B30] Rimm EB Chan J Stampfer MJ Colditz GA Willett WC Prospective study of cigarette smoking, alcohol use, and the risk of diabetes in men BMJ 1995 310 6979 555 559 10.1136/bmj.310.6979.555 7888928 PMC2548937

[B31] Manson JE Ajani UA Liu S Nathan DM Hennekens CH A prospective study of cigarette smoking and the incidence of diabetes mellitus among US male physicians Am J Med 2000 109 7 538 542 10.1016/S0002-9343(00)00568-4 11063954

[B32] Fagard RH Nilsson PM Smoking and diabetes--the double health hazard Prim Care Diabetes 2009 3 4 205 209 10.1016/j.pcd.2009.09.003 19875348

[B33] Ceriello A Bortolotti N Motz E Crescentini A Lizzio S Russo A Tonutti L Taboga C Meal-generated oxidative stress in type 2 diabetic patients Diabetes Care 1998 21 9 1529 1533 10.2337/diacare.21.9.1529 9727904

[B34] Marfella R Quagliaro L Nappo F Ceriello A Giugliano D Acute hyperglycemia induces an oxidative stress in healthy subjects J Clin Invest 2001 108 4 635 636 11518739 10.1172/JCI13727 PMC209408

[B35] Tomiyama H Kimura Y Okazaki R Kushiro T Abe M Kuwabara Y Yoshida H Kuwata S Kinouchi T Doba N Close relationship of abnormal glucose tolerance with endothelial dysfunction in hypertension Hypertension 2000 36 2 245 249 10.1161/01.HYP.36.2.245 10948085

[B36] Kawano H Motoyama T Hirashima O Hirai N Miyao Y Sakamoto T Kugiyama K Ogawa H Yasue H Hyperglycemia rapidly suppresses flow-mediated endothelium-dependent vasodilation of brachial artery J Am Coll Cardiol 1999 34 1 146 154 10.1016/S0735-1097(99)00168-0 10400004

[B37] Heitzer T Schlinzig T Krohn K Meinertz T Munzel T Endothelial dysfunction, oxidative stress, and risk of cardiovascular events in patients with coronary artery disease Circulation 2001 104 22 2673 2678 10.1161/hc4601.099485 11723017

[B38] Haenni A Andersson PE Lind L Berne C Lithell H Electrolyte changes and metabolic effects of lisinopril/bendrofluazide treatment. Results from a randomized, double-blind study with parallel groups Am J Hypertens 1994 7 7 Pt 1 615 622 7946163 10.1093/ajh/7.7.615

